# Gastric Outlet Obstruction as the Initial Presentation of Upper Tract Urothelial Carcinoma

**DOI:** 10.1155/2020/8850062

**Published:** 2020-10-19

**Authors:** Karl Andersen, Sarah Burroughs, Assad Munis, Ryan T. Hoff, Alan Shapiro

**Affiliations:** ^1^Advocate Lutheran General Hospital, 1775 Dempster Street, Park Ridge, IL 60068, USA; ^2^Department of Internal Medicine, Division of Gastroenterology, Advocate Lutheran General Hospital, 1775 Dempster Street, Park Ridge, IL 60068, USA

## Abstract

GOO is often the first sign of advanced upper gastrointestinal neoplasms. The most common neoplasms associated with GOO include gastric, pancreatic, and biliary tract cancers. Urinary tract urothelial carcinoma has been a rarely documented cause of GOO.

## 1. Introduction

Gastric outlet obstruction (GOO) is a syndrome characterized by abdominal pain and vomiting secondary to mechanical obstruction. GOO is often the first sign of advanced upper gastrointestinal neoplasms, either as a result of direct invasion into the lumen of the digestive system or by external compression. Symptoms of obstruction are often vague and include early satiety, weight loss, gastroesophageal reflux disease (GERD), and abdominal pain [1]. Causes of GOO include peptic ulcer disease, gastric polyps, infiltrative disease, and malignancy. The most common neoplastic causes of GOO include gastric, pancreatic, and biliary tract cancers [2]. Locally invasive upper tract urothelial carcinoma (UTUC) is a rare cause of malignant GOO. This case report demonstrates the potential difficulty in diagnosing this disease and the need for a multidisciplinary approach.

## 2. Case Presentation

An 80-year-old female initially presented to her primary care physician's office with three weeks of right-sided flank pain. Her past medical history was notable for GERD, chronic constipation, and Barrett's esophagus. Her initial physical exam was significant for tenderness to palpation in the right lower quadrant of her abdomen, and was otherwise unremarkable. A computed tomography (CT) scan of her abdomen and pelvis showed dilatation of the renal pelvis, a thickened and dilated terminal ileum, and inflammatory changes with increased tissue around the right ureter. She was evaluated by urology and underwent a cystoscopy, which showed a ureteral stricture that was unable to be traversed. A ureteral stent was placed across the stricture. Urine cytology and ureteral brushings were negative for malignancy. Her symptoms modestly improved. Two months later, she continued to report right-sided flank pain despite stent placement. She underwent removal of right ureteral stent, ureteroscopy, and pyelogram which showed stable narrowing of the ureter in addition to dilatation of the upper and lower pole calices from prior evaluation. She was instructed to follow up in 6–8 weeks for diuretic renal scan.

**Figure 1:**
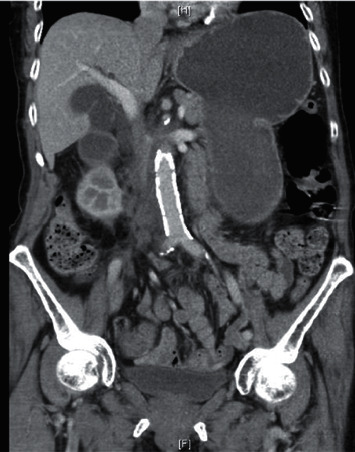
Coronal view of CT scan of the abdomen and pelvis showing wall thickening of the 2nd and 3rd parts of the duodenum and proximal distention of the 1st part of the duodenum and stomach.

**Figure 2:**
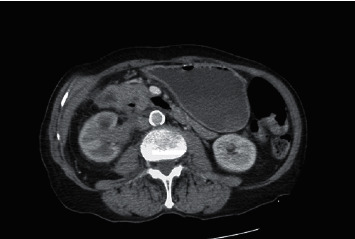
Axial view of the CT scan of the abdomen and pelvis showing a mass in the right renal pelvis.

**Figure 3:**
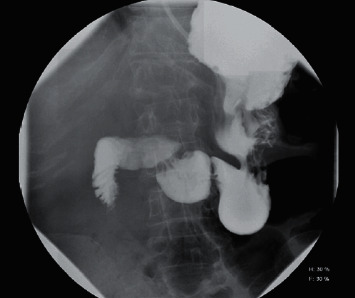
X-ray small bowel follow-through partial obstruction in the 2^nd^ portion of the duodenum as evidenced by narrowing without contrast follow-through.

Two weeks later, she presented with five days of diffuse abdominal pain and nonbloody, nonbilious emesis. She denied constipation or diarrhea but did report a lack of flatulence. Her physical exam was significant for diffuse, mild abdominal tenderness. Laboratory studies showed a serum white blood cell count of 11.7/mcL, and a urinalysis with moderate leukocyte esterase and moderate bacteria. Liver enzymes, lipase, and bilirubin were normal. A CT scan of the abdomen and pelvis with intravenous contrast showed an increase in the mass-like thickening of the right renal pelvis measuring 3.1 × 2.3 cm in size and partially encasing the renal artery and vein. It extended into the retroperitoneum resulting in compression of the duodenum. New gallbladder distention and biliary ductal dilatation was noted, with the common bile duct measuring 1.2 cm in diameter (Figures 1 and 2). A small bowel follow-through confirmed partial obstruction of the second portion of the duodenum (Figure 3). A CT-guided core and fine needle aspiration biopsy was then performed of the mass, revealing poorly differentiated upper tract urothelial carcinoma (UTUC) with immunohistochemical stains positive for CK-7, p63, and GATA3, and negative for PAX-8 and CK-20.

A gastrojejunostomy tube was inserted for nutrition and gastric decompression. She was evaluated by medical oncology and successfully completed four cycles of gemcitabine and cisplatin, which led to a decrease in mass size and resolution of the GOO, allowing for removal of the gastrojejunostomy tube. She then underwent a right nephroureterectomy followed by four more cycles of chemotherapy. Four months later, she was readmitted for small bowel obstruction secondary to peritoneal metastasis. A percutaneous endoscopic gastrostomy tube was inserted for decompression; however, the patient continued to decline and she was ultimately enrolled in hospice.

## 3. Discussion

Few cases of UTUC as the cause of GOO have been reported [1, 3]. Urothelial carcinoma (UC), also known as transitional cell carcinoma, is the most common cause of bladder cancer [4]. UTUC is a rare form of UC and arises from the renal pelvis and ureter. It makes up approximately 5–10% of all UCs with an incidence of about 3,500 cases per year [5]. UTUC spreads more commonly by direct invasion and extension and through the lymphatic system, unlike UC from the bladder. The thin ureteral walls may allow direct extension of UTUC tumors to occur more commonly than with bladder malignancies [6]. Diagnosis is usually made through a combination of cross-sectional imaging, ureteropyeloscopy with cystoscopy, brush biopsy, and urine cytology. Brush biopsy, as proved to be the case here, has a higher rate of false-negative results in cases of poorly differentiated UTUC compared to well-differentiated UTUC [7].

About 85% of patients with newly diagnosed urothelial carcinomas present with gross or microscopic hematuria occurring in the majority of patients [8]. However, more advanced disease may present in an atypical fashion, as outlined in this case. GOO is a clinical and pathophysiological syndrome as a consequence of a disease process resulting in mechanical obstruction. The most common malignant cause of GOO occurs when a gastrointestinal cancer causes intraluminal obstruction. Pancreatic cancer is the most common cause of obstruction, but other primary gastrointestinal cancers, such as gastric, duodenal, ampullary, and cholangiocarcinoma, have been documented. To our knowledge, this is the third case of UTUC causing GOO, and it demonstrates the challenge diagnosing UTUC can pose. It should also serve as a reminder that a broad differential should be entertained when determining the etiology of GOO.
